# Neuroendocrine tumors presenting with thyroid gland metastasis: a case series

**DOI:** 10.1186/1752-1947-6-73

**Published:** 2012-02-27

**Authors:** Emre Sivrikoz, Nese Colak Ozbey, Bulent Kaya, Yesim Erbil, Serkan Kaya, Dilek Yilmazbayhan, Pinar Firat, Yersu Kapran

**Affiliations:** 1 Department of General Surgery, Istanbul University, Istanbul Medical School, Capa-Istanbul 34093, Turkey; 2 Department of Internal Medicine, Istanbul University, Istanbul Medical School, Capa-Istanbul 34093, Turkey; 3 Department of Thoracic Surgery, Istanbul University, Istanbul Medical School, Capa-Istanbul 34093, Turkey; 4 Department of Pathology, Istanbul University, Istanbul Medical School, Capa-Istanbul 34093, Turkey

## Abstract

**Introduction:**

Autopsy series have shown that metastasis to the thyroid gland has occurred in up to 24% of patients who have died of cancer. Neuroendocrine tumors may metastasize to thyroid gland.

**Case presentations:**

Case 1 was a 17-year-old Turkish woman who was referred from our Endocrinology Department for a thyroidectomy for treatment of neuroendocrine tumor metastasis. She was treated with a bilateral total thyroidectomy. Histopathological examination results were consistent with a neuroendocrine tumor; neoplastic cells showed strong immunoreactivity to chromogranin A and synaptophysin, but the immunohistochemical profile was inconsistent with medullary thyroid carcinoma in that the tumor was negative for calcitonin, carcinoembryonic antigen, and thyroid transcription factor-1.

Case 2 was a 54-year-old Turkish woman who presented with a 3-cm nodule on her right thyroid lobe. She had undergone surgery for a right lung mass four years previously. After a right pneumonectomy, thymectomy and lymph node dissection, a typical carcinoid tumor was diagnosed. Under ultrasonographic guidance, fine needle aspiration biopsy of her right thyroid pole nodule was performed and the biopsy was compatible with a neuroendocrine tumor metastasis. She was treated with a bilateral total thyroidectomy. Histopathological examination indicated three nodular lesions, 5 cm and 0.4 cm in diameter in her right lobe and 0.1 cm in diameter in her left lobe. The tumors were consistent with a neuroendocrine phenotype, showing strong immunoreactivity to chromogranin A and synaptophysin.

**Conclusion:**

Thyroid nodules detected during follow-up of neuroendocrine tumor patients should be thoroughly investigated. A fine needle aspiration biopsy of the thyroid confirms the diagnosis in most cases and leads to appropriate management of those patients and may prevent unnecessary treatment approaches.

## Introduction

Neuroendocrine tumors may cause paraneoplastic syndromes by secreting endogenous hormone-active substances. The resulting symptoms may mask the primary tumor and cause a delay in diagnosis, which eventually leads to metastatic disease. During the disease course, a thyroid metastasis mimicking medullary carcinoma may rarely develop, making the diagnosis even more complicated. Two cases of thyroid metastasis from neuroendocrine tumors are described in this paper, with a discussion of their challenging diagnostic features and subsequent treatment.

## Case presentations

### Case 1

A 17-year-old Turkish female patient was referred from our endocrinology department for a thyroidectomy to treat a neuroendocrine tumor metastasis. Our patient had presented to a university hospital 30 months previously, with dysmenorrhea, hirsutism and facial swelling. Her symptoms were investigated and she was suspected of having ectopic Cushing's syndrome. At that time, magnetic resonance imaging (MRI) of her sella turcica was performed, with normal findings. Her 24-hour urinary free cortisol level was 159 μg/day, and her basal adrenocorticotropic hormone (ACTH) concentration was 80 pg/mL. She was referred to our endocrinology department one month later and the investigations were repeated. Biochemical findings indicated ACTH-dependent Cushing's syndrome. Her ACTH and 24-hour urinary free cortisol concentrations were 106 pg/mL and 240 μg/day respectively. A high-dose (8 mg) dexamethasone suppression test revealed non-suppressible cortisol concentrations. Cushing's syndrome due to ectopic ACTH secretion was suspected. An ultrasonagraph (USG) of her thyroid revealed normal findings. There was no abnormality on thoracic computed tomography (CT) and on adrenal MRI findings.

Her serum calcitonin levels were within the normal range. Her 24-hour urine metanephrines and 5-hydroxyindoleacetic acid (5-HIAA) concentrations were within their respective normal ranges. Sampling of her inferior petrosal sinus was performed and revealed a central-to-periphery ACTH ratio of below 1.8, which indicates ectopic ACTH secretion. Octreotide scintigraphy was performed, which revealed scarce activity in her upper mediastinum.

Close follow-up was planned for this patient; however, she did not attend her appointments. Two years later, in August 2009, she presented with worsening of her initial symptoms. MRI of her abdomen and sellar was performed, demonstrating normal findings. A USG of her thyroid detected a 1 cm nodule at the right lower pole, and fine needle aspiration (FNA) revealed non-diagnostic material. Her ACTH concentrations were high (153 pg/mL) and her 24-hour urinary cortisol concentration was 2454 μg/day. Her thyroid function test results were within normal limits. A thoracic CT demonstrated mediastinal lymphadenopathies (LAPs). She was referred to our thoracic surgery department and a mediastinoscopy was performed.

Histopathological evaluation revealed a metastatic neuroendocrine tumor on her lymph nodes. Positron emission tomography-CT revealed hypermetabolic LAPs in her right supraclavicular, left prevascular mediastinal, right upper paratracheal and anterior mediastinal regions and disseminated bony metastases. The primary tumor site was not diagnosed. Her urinary 5-HIAA was within normal limits (2.88 mg/day; normal range: 2.0 mg/day to 9.0 mg/day). Bone scintigraphy revealed pathological activities at the superior portion of her medial left scapular border; her thoracic vertebrae T7, T10 and T11; her right sacroiliac joint; her inferior right acetabular region; and the posterior portion of her right eighth rib, indicating metastases.

Our patient was started on 30 mg of octreotide long-acting release (LAR) every 28 days with the aim of controlling her ectopic ACTH secretion and tumor progression. A reoperation was planned to remove the mediastinal masses. Excision of the mediastinal mass, mediastinal LAP and right supraclavicular LAP was performed via upper sternotomy. Histopathological and immunohistochemical analyses revealed a metastatic atypical carcinoid tumor with positive ACTH immunostaining and a 10% Ki-67 labeling index. After the operation, an amelioration of the signs and symptoms of Cushing's syndrome was observed and our patient's basal ACTH concentration decreased to 88.6 pg/mL. She remained on the octreotide-LAR treatment.

**Figure 1:**
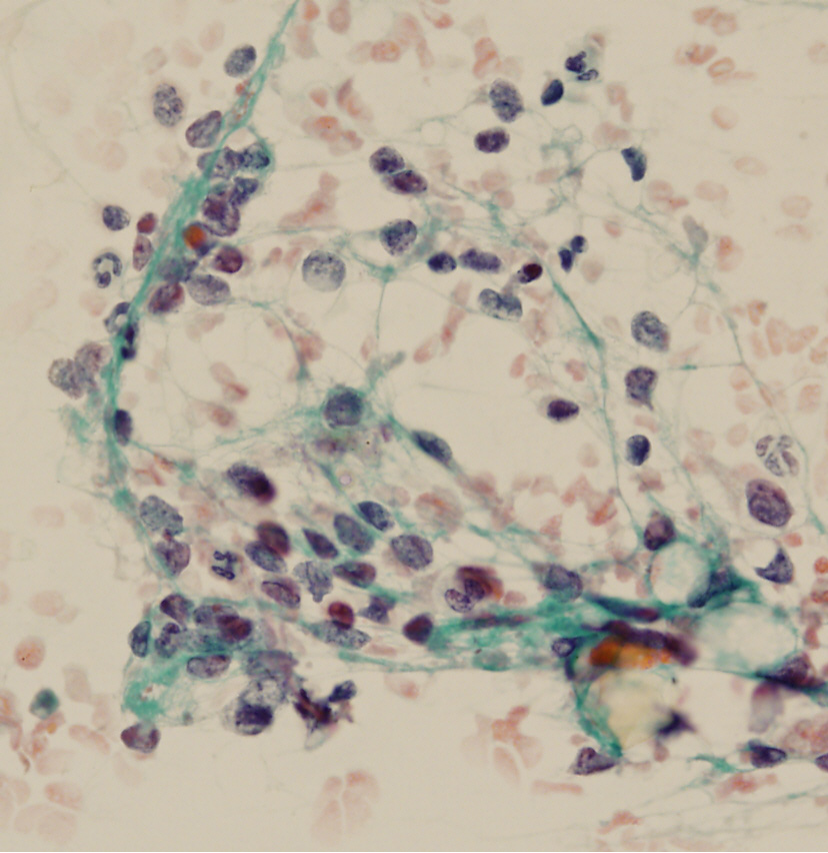
**Case 1: singly distributed neoplastic cells showing salt-and-pepper chromatin consistent with a neuroendocrine neoplasm (×60 Papanicolaou stain)**.

One month after surgery, a repeated thyroid USG showed that the previously detected thyroid nodule had increased in size (15 mm at its maximum diameter). Under USG guidance, FNA of her right thyroid pole nodule was performed, and the aspirate was compatible with a neuroendocrine tumor metastasis. Our patient was treated with a bilateral total thyroidectomy. Gross examination of the thyroidectomy specimen demonstrated a solitary nodule 1.8 cm × 1.4 cm × 0.8 cm in diameter. Histopathology results were consistent with a neuroendocrine tumor. Neoplastic cells showed strong immunoreactivity to chromogranin A and synaptophysin, but the immunohistochemical profile was inconsistent with medullary thyroid carcinoma in that the tumor was negative for calcitonin, carcinoembryonic antigen (CEA), and thyroid transcription factor-1 (TTF-1) (Figure 1).

Our patient is still being followed up at our institution. Her metastatic lesions are stable on octreotide LAR 30 mg/day. However, progressive deterioration in signs and symptoms of Cushing's syndrome (that is, glucose intolerance, hypertension, myopathy and osteoporosis) led us to determine that a bilateral adrenalectomy should be performed on this patient.

### Case 2

**Figure 2:**
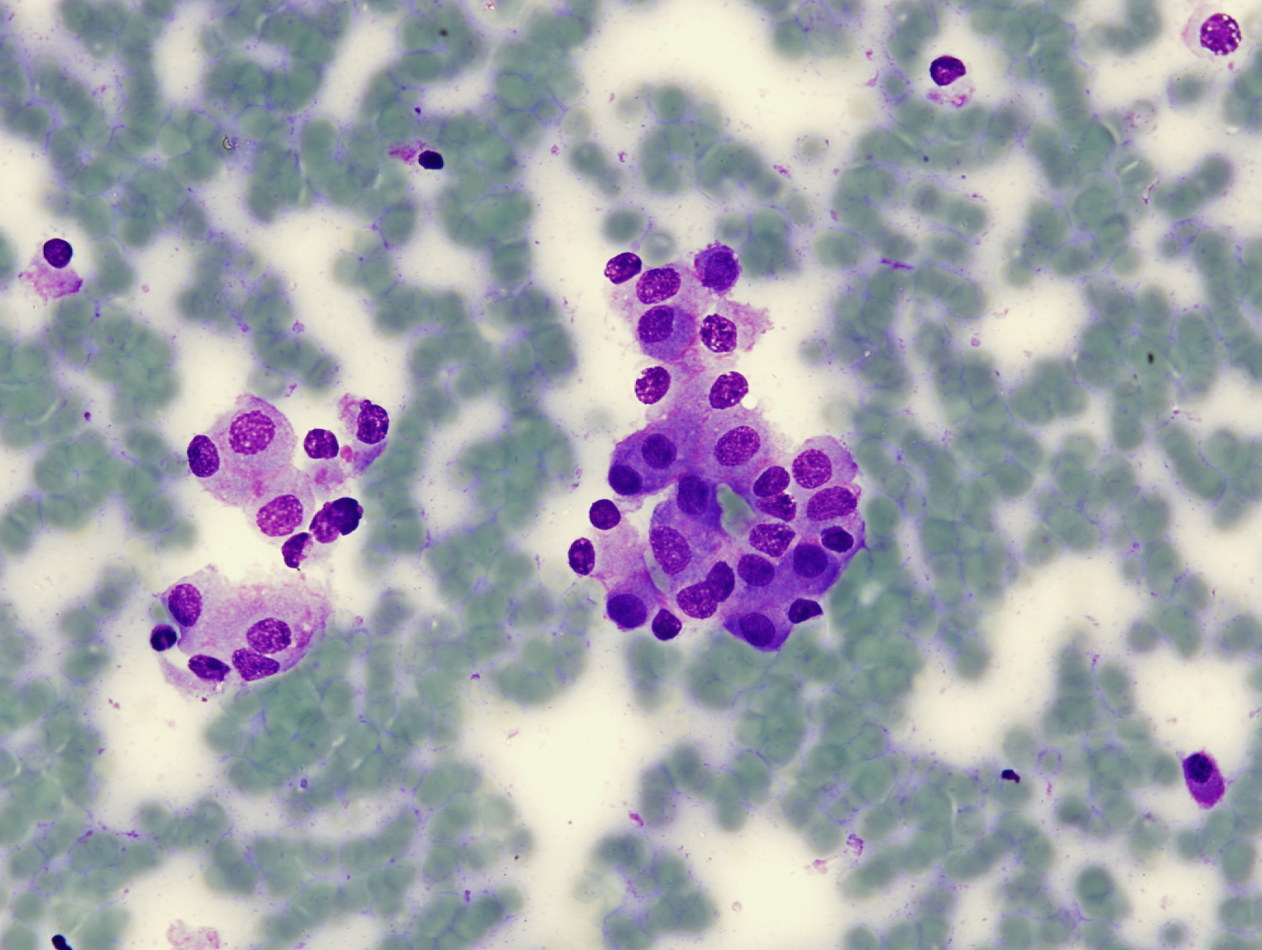
**Case 2: plasmacytoid monotonous epithelial cells forming loose groups compatible with a neuroendocrine neoplasm (×60; May-Grünwald-Giemsa stain)**.

A 54-year-old Turkish woman presented with a 3 cm nodule on her right thyroid lobe. She had undergone surgery for a right lung mass in February 2007. After a right pneumonectomy, thymectomy and lymph node dissection, a typical carcinoid tumor was diagnosed and she was maintained on regular follow-up. Her serum calcitonin was within the normal range. Ectopic hormone syndrome was not identified. Under USG guidance, FNA of her right thyroid pole nodule was performed, and the biopsy was compatible with a neuroendocrine tumor metastasis. She was treated with a bilateral total thyroidectomy. A histopathological examination indicated three nodular lesions, 5 cm and 0.4 cm in diameter in the right lobe and 0.1 cm in diameter in the left lobe. The tumors were consistent with a neuroendocrine phenotype; showing strong immunoreactivity to chromogranin A and synaptophysin, but the immunohistochemical profile was inconsistent with medullary thyroid carcinoma in that the tumor was negative for calcitonin, CEA and TTF-1 (Figure 2).

## Discussion

Although the thyroid gland has an affluent blood supply, solid tumor metastases to the thyroid are seldom reported during follow-up. Lin *et al. *[1] reported that 14 of 1,013 patients (1.4%) with thyroid cancer verified on histology had metastatic tumors; adenocarcinoma and squamous cell carcinoma were the most frequently detected histological types [1]. Results of autopsy series have indicated that metastases to the thyroid gland have been found in up to 24% of patients who have died of cancer [1,2]. Therefore, in most cases, thyroid metastasis remains clinically silent.

Metastases to the thyroid should be a sign of more widespread disease. Lin *et al. *[1] reported that most of their patients with thyroid metastases died within nine months of diagnosis. However, in some slowly growing tumors, a relatively longer survival is still possible, as observed in our first patient. Aggressive tumors lead to painful thyroid nodules with rapid growth and local compression symptoms. Thyrotoxicosis due to destructive thyroiditis has also been described [3,4]. In both of our patients, thyroid function test results were within normal limits and local symptoms were not evident. The slow growth of well-differentiated neuroendocrine tumors could be responsible for the absence of thyroid destruction and local symptoms. Thyroid metastases were detected two and three years after the diagnosis of neuroendocrine tumors in our first and second patients, respectively. Lam and Lo [5] reported that the mean latency period between diagnosis of primary tumors and thyroid metastasis was nine months in their series.

In most cases, breast, lung, stomach and renal cell carcinomas are responsible for metastatic thyroid lesions [1,2,5]. To the best of our knowledge, seven thyroid metastases of neuroendocrine tumors have previously been reported [6-12]. Matias-Guiu *et al. *[13] reported an additional six patients in whom the diagnosis was confirmed by their immunohistochemical profile and morphologic findings of tumor tissue. When the diagnosis of a primary tumor is not apparent, thyroid metastases of neuroendocrine carcinoma may lead to diagnostic confusion. Metastatic thyroid lesions from neuroendocrine tumors may mimic medullary thyroid carcinoma on cytology or on histopathology [7,10,13]. Immunohistochemical markers such as chromogranin A, neuron-specific enolase and synaptophysin are reportedly positive for both medullary thyroid and metastatic neuroendocrine carcinoma [11,12]. Calcitonin and CEA immunohistochemistry should be negative in metastatic neuroendocrine tumors, but positive staining is observed in medullary thyroid cancer [7,11,12]. Calcitonin immunostaining may even be positive in neuroendocrine tumors [12,14]. In particular, the small cell variant of medullary thyroid carcinoma has been reported to resemble small cell pulmonary neuroendocrine carcinoma [15]. In our patients, the previous non-thyroidal neuroendocrine carcinoma diagnosis and normal serum calcitonin concentrations were adequate for accurate diagnosis in addition to the histopathological and immunohistochemical findings of thyroid metastases.

Neuroendocrine tumors may present with paraneoplastic syndromes, which may mask the primary lesion. Our first patient had ectopic ACTH syndrome, which was responsible for her comorbidities. Bronchial carcinoid tumors are reportedly the most frequent cause of ectopic ACTH secretion [16]. Despite a stable metastatic disease, our patient had progressively worsening signs and symptoms of Cushing's disease. Therefore, a bilateral adrenalectomy was considered. Somatostatin analogs have been used for the treatment of ectopic ACTH secretion with varied success rates [17,18]. During long-term treatment, antiproliferative and antisecretory effects of octreotide treatment for endocrine tumors may dissociate, leading to sustained antiproliferative but lessened antisecretory effects [19]. In our first patient, we believe that the antisecretory effects of octreotide LAR have diminished but that the antiproliferative effects are still obvious because no considerable increase in the size of her metastatic lesions has been observed.

Different mechanisms have been proposed to explain the resistance to the antisecretory effects of octreotide during treatment, such as different expression patterns of somatostatin receptor subtypes [19,20]. On the other hand, an increase in the mass of secondary deposits under the detection limit of radiologic imaging techniques could be responsible for the exacerbation of Cushing's syndrome characteristics in our first patient. An increase in the dose of somatostatin analogs may overcome this resistance. Alternatively, medical or chemical adrenalectomy should be considered.

## Conclusion

It is important to consider the possibility of metastasis in patients with thyroid nodules and a history of a primary malignant tumor. Thyroid nodules detected during follow-up of patients with neuroendocrine tumors should be thoroughly investigated. Thyroid fine-needle aspiration biopsy confirms the diagnosis in most cases, leads to appropriate management of these patients, and may prevent unnecessary treatment approaches.

## Consent

Written informed consent was obtained from the patient's legal guardian (Case 1) and from the patient (Case 2) for publication of this case series and any accompanying images. Copies of the written consent are available for review by the Editor-in-Chief of this journal.

## Competing interests

The authors declare that they have no competing interests.

## Authors' contributions

ES, NCO and YE collected the information regarding the case. SK was the thoracic surgeon. ES completed the literature review and wrote the manuscript. YE and BK contributed to writing the manuscript. DY, PF and YK were the pathologists. All authors have read and approved the final manuscript.
